# Lithium enhances exercise-induced glycogen breakdown and insulin-induced AKT activation to facilitate glucose uptake in rodent skeletal muscle

**DOI:** 10.1007/s00424-021-02543-0

**Published:** 2021-03-03

**Authors:** Su-Ryun Jung, Sol-Yi Park, Jin-Ho Koh, Jong-Yeon Kim

**Affiliations:** 1 College of Pharmacy, Keimyung University, Daegu, Republic of Korea; 2 Department of Physiology, College of Medicine, Yeungnam University, Gyeongsan, Republic of Korea

**Keywords:** Lithium, Exercise, T1DM, Glycogen breakdown, GSK3β, AKT

## Abstract

The purpose of this study was to investigate the effect of lithium on glucose disposal in a high-fat diet-induced type 2 diabetes mellitus (T2DM) and streptozotocin-induced type 1 diabetes mellitus (T1DM) animal model along with low-volume exercise and low-dose insulin. Lithium decreased body weight, fasting plasma glucose, and insulin levels when to treat with low-volume exercise training; however, there were no adaptive responses like an increase in GLUT4 content and translocation factor levels. We discovered that lithium enhanced glucose uptake by acute low-volume exercise-induced glycogen breakdown, which was facilitated by the dephosphorylation of serine 473-AKT (Ser473-AKT) and serine 9-GSK3β. In streptozotocin-induced T1DM mice, Li/low-dose insulin facilitates glucose uptake through increase the level of exocyst complex component 7 (Exoc7) and Ser473-AKT. Thus, lithium enhances acute exercise-induced glycogen breakdown and insulin-induced AKT activation and could serve as a candidate therapeutic target to regulate glucose level of DM patients.

**Supplementary Information:**

The online version contains supplementary material available at 10.1007/s00424-021-02543-0.

## Introduction

Approximately 50 years ago, lithium, an alkali metal element was found to increase glucose uptake and glycogen synthesis in rat diaphragm muscle [12, 21], similar to the effects of insulin and contraction on skeletal muscle and adipose tissue; consequently, lithium chloride (Li) has become the most commonly administered medicine for mania patients [5, 8, 13, 21]. Li is not considered an attractive molecule for the development of a clinical treatment, as its effect is significantly lower than that of insulin or muscle contraction [37], and further research is still required.

Glucose uptake induced by muscle contraction or insulin is regulated by two different underlying mechanisms; however, both the mechanisms use glucose transporter 4 (GLUT4) for glucose uptake. Signaling due to muscle contraction and insulin mediates the translocation of GLUT4 to the plasma membrane and activates GLUT4-induced glucose uptake. A previous study indicated that Li induces GLUT4 translocation to the plasma membrane, but GLUT4 is not much activated to take up glucose into the cellular than insulin and exercise [24], and the amount is only 30–40% of the rate of glucose uptake induced by insulin or tetanic contraction. This result is due to the distinct mechanisms underlying translocation and activation of GLUT4 [15, 22]; thus, Li is known to mediate GLUT4 translocation to the plasma membrane; however, the other effects of Li on glucose uptake induced by muscle contraction and insulin remain unknown.

In addition to GLUT4 translocation, various distinct mechanistic signaling pathways induced by muscle contraction or insulin are involved in glucose uptake. Previous studies have shown that low glycogen levels induced by muscle contraction play a key role in glucose uptake, and insulin-mediated protein kinase B (PKB; also known as AKT) activation also induces GLUT4 translocation and stimulates glucose uptake [36]. Although many signaling cascades are mediated by muscle contraction or insulin induce glucose uptake, GLUT4 translocation and activation is an essential step for the uptake of glucose into the skeletal muscle. Li can strongly mediate GLUT4 translocation, even with weak activation [24], indicating that it could be a candidate or supporter to facilitate glucose uptake due to another activator, such as muscle contraction or insulin. Generally, high-fat diet (HFD)-induced insulin resistance (IR) is caused by the reduction of GLUT4 translocation through a defective insulin signaling pathway [39], and in type 1 diabetes mellitus (T1DM) patients, the translocation and activation of GLUT4 does not occur due a lack of insulin production. Since Li can only enhance GLUT4 translocation to the plasma membrane, we speculated that if a low dose of insulin or muscle contraction could be used to activate GLUT4, Li-induced GLUT4 translocation may take up glucose sufficiently in the isolated skeletal muscle and improve glucose homeostasis that is dysregulated in IR and T1DM patients who cannot actively exercise or use high-dose insulin; since Li is already used in the clinic, and its metabolic side effects are hard to find except for the contradictory results on the glomerulus [9, 28, 30, 31, 40]; thus, Li could serve as a promising alternative.

A previous study has only determined Li effect in vitro [24]; therefore, in the current study, we investigated the significance of Li effect on glucose disposal in a HFD-induced T2DM and streptozotocin-induced T1DM animal model along with low-volume exercise and low-dosage insulin, which were not sufficient to stimulate glucose uptake.

## Methods

### HFD-induced T2DM rats

Male Sprague-Dawley rats (8 weeks old; *n* = 50) were fed an HFD for 8 weeks to make diet-induced obese (DIO), and 10 animals were excluded as a chow control group. After 8 weeks, rats were randomly divided for 4 groups (fat control; FC, Li; fat-lithium, Ex; fat-exercise training, LiEx; fat-Li + exercise training, *n* = 10 each). Rats were provided chow (carbohydrate 67%, fat 13%, protein 20%, Purina, USA) or HFD (carbohydrate 42.7%, fat 42%, protein 15.2%, Purina, USA) with water ad libitum. During the course of the experiment, body weight, and food consumption were measured every 2 days. The study design was approved by the Animal Experiment Ethics Committee of Daegu Technopark BioHealth Convergence Center (BHCC-IACUC-2018-01).

Ten milligram per kilogram Li chloride (L4408, Sigma-Aldrich, St. Louis, MO, USA) was administered orally once a day, 5 days a week for 12 weeks. Previous studies have shown that 10 mg/kg Li induces 0.3–0.7 mmol/L in blood that is recommended amount for the treatment of psychiatric diseases [16, 38, 45]. We tested toxicity and found that long-term Li treat did not induce toxicity in the kidney and liver of DIO rats (Fig. S1). In LiEx group, Li was gavage 1 hour before exercise training. The control group was orally administered the same amount of saline. Exercise intensity was set lower than the lactate threshold to avoid masking the Li effect due to strong exercise stimulation [43]. The exercise protocol consisted of low intensity (40% VO2 max, 12 m/min, slop 0%) walking on a treadmill (FT-200, Techman Soft) for 20 min per day, 3 days a week for 12 weeks.

After 12 weeks of treatment, rats were rested for 48 h to eliminate the last-bout exercise effect. The rats were fasted overnight, following which they were anesthetized with pentobarbital sodium (5 mg/100 g bwt), body composition was measured using DEXA, and the tissues were removed. The extracted liver and kidney were fixed with 4% formalin solution for hematoxylin and eosin (H&E) staining. The plantaris muscles were clamp frozen after dissection and stored at – 80 °C until analysis. The abdominal cavity was opened to collect 5 ml of blood from the abdominal artery, which was anticoagulated with 50 μl of heparin to prevent clotting. After centrifugation (1500×*g*, 15 min), only plasma was stored at – 80 °C.

### Streptozotocin-induced T1DM mice

T1DM was induced in male C57BL6/J mice (10 weeks, *n* = 25) by intraperitoneal injection with 50 mg/kg streptozotocin (#S-0130, Sigma-Aldrich), because no difference in glucose metabolism between rats and mice [1, 27, 41], and it is more efficient to make T1DM with mice rather than rats. After 2 weeks, mice with fasting blood glucose concentrations of ≥ 350 mg/dL were selected as the streptozotocin-induced diabetic mice [26]. After 3 days of Li administration, the mice were divided into the following four groups: control; CC, lithium; Li, low-dose insulin; LDI, Li + low-dose insulin; Li/LDI, Li + low-volume exercise; Li/Low-Ex, Li + moderate-volume exercise; Li/Mo-Ex (*n* = 5 per group). Fasting for 6 h, Li was administered orally, then 1 h later, insulin or exercise was treated. The insulin group was injected with 0.1 U/kg insulin (Humulin R U-100) intraperitoneally. The exercise group was subjected to treadmill walking/running at a low (12 m/min, slope 0%, 20 min) or moderate (18 m/min, slope 0%, 30 min) volume. Immediately after treatment, the animals were anesthetized with sodium pentobarbital (5 mg/100 g body weight) and plantaris muscles were removed then used to measure muscle glycogen content and the rest of them used to western blotting.

### Glucose transport rate in skeletal muscles

Male Wistar rats (∼ 125 g) were fasted for 12 h, then they were anesthetized with pentobarbital sodium (5 mg/100 g body wt). Thereafter, epitrochlearis muscle was excised and incubated in Dubnoff shakers in 25-ml Erlenmeyer flasks containing 2 ml of incubation media consisting of 0.1% radioimmunoassay grade bovine serum albumin (BSA) in Krebs-Henseleit bicarbonate buffer (KHB) [17] with sufficient mannitol to maintain constant osmolarity. Flasks were gassed with 5% CO_2_–95% O_2_. Muscles were allowed to recover for 30 min after dissection at 35 °C with 2 mM sodium pyruvate. Muscles were preincubated with or without 10 mM Li (Sigma-Aldrich), insulin (Pork insulin, Eli Lily, USA), or anisomycin (Sigma-Aldrich, A9789) for 60 min (35°C). For insulin stimulation, three insulin concentrations were used (physiological con. 60 μU/ml, maximum con. 2 m U/ml, and low con. 20 μU/ml) [18, 19]. The muscle contraction was induced by treatment with 9~19 mM anisomycin. The concentration of Li was maintained at 10 mM, which is the concentration that resulted in the maximum glucose transport rate in previous studies [37]. To eliminate glucose from extracellular tissues after incubation, muscle samples were washed for 10 min with KHB containing 40 mM mannitol at 30 °C. After washing, the samples were incubated at 30 °C for 20 min (second incubation) in KHB containing 4 mM 2[^1,3-3^H]DG (1.5 μCi/ml, American Radiolabeled Chemicals) and 36 mM[^14^C] mannitol (0.2 μCi/ml, ICN Radiochemicals). Same stimuli as in preincubation were maintained during washing and second incubation. The samples were compress-frozen and stored at – 80 °C until analysis. The intracellular and extracellular concentrations of ^3^H and ^14^C were measured with scintillation counters [20].

### Blood profile and muscle glycogen

Blood glucose levels were determined using a glucose analyzer (YSI 2300, Springfield, USA), ELISA kits for determining insulin (Mercodia, Uppsala, Sweden), AST, and ALT (Cayman tech., USA) levels were purchased and used according to the manufacturers’ protocols. Muscle glycogen level was estimated using a glycogen assay kit (Abcam, USA).

### H&E staining

The liver and kidney were fixed in 4% formalin solution for 2 days and cut into cross sections for observation. The vertical section was embedded with paraffin to produce paraffin blocks. This block was cut at 4 μm thickness and attached on a glass slide. Samples were observed with H&E staining, which was performed using a deparaffinization and hydration process with xylene and alcohol. A virtual file was produced from these slides using Scan scope XT (Aperio Technologies, CA92081), while the average value of the cross-sectional area of the muscle fiber was analyzed using the Image scope program (Aperio Technologies, version 10.2.2.2319).

### Western blotting

The extracted plantaris muscle was homogenized using ice-cold RIPA buffer comprising 250 mM sucrose, 10 mM HEPES/1 mM ethylenediaminetetraacetic acid (EDTA, pH 7.4), 1 mM Pefabloc (Roche), 1 mM EDTA, 1 mM NaF, 1 μg/ml aprotinin, 1 μg/ml leupeptin, 1 μg/ml pepstatin, 0.1 mM bpV (phen), and 2 mg/ml glycerophosphate. The homogenized sample was subjected to three freeze/thaw cycles and centrifuged (700×*g*, 15 min). After quantifying the protein concentration, the prepared sample was dissolved in Laemmli buffer, loaded onto a sodium dodecyl sulfate-polyacrylamide gel, electrophoresed, and transferred to a nitrocellulose membrane. The membrane was blocked for 60 min using 5% non-fat dry milk and Tris-buffered saline + 0.1% Tween 10 (TBST; pH 7.5), washed with TBST, and incubated overnight at 4 °C with primary antibodies against the following: GLUT4 (Santa Cruz Bio, sc-53566), GSK3β, phospho-GSK3β (Santa Cruz Bio, sc-81462, sc-373800), Exoc7(Santa Cruz Bio, sc-365825), Rab10 (Santa Cruz Bio, sc-101429), Dynamin (Santa Cruz Bio, sc-17807), phospho-AKT (Cell signaling, 4060), AKT(Cell signaling, 2920), and β-actin (abcam, ab106814). After washing with TBST, the samples were treated with the secondary antibody (anti-mouse or anti-rabbit, Santa Cruz Biotechnology, Santa Cruz, CA, USA) for 60 min. The bands were visualized using ECL (Genekhan Scientific, St. Louis, MO, USA), and the relative intensity of the bands was assessed using SigmaGel (Jandel Scientific Corp., Erkrath, Germany).

### Statistical analysis

Values are means ± SE. The significance of the differences between groups was assessed using a one-way analysis of variance followed by post hoc comparison using the Tukey significant difference method.

## Results

### Long-term Li and Ex training improve body composition and blood glucose level in DIO rats

**Fig. 1:**
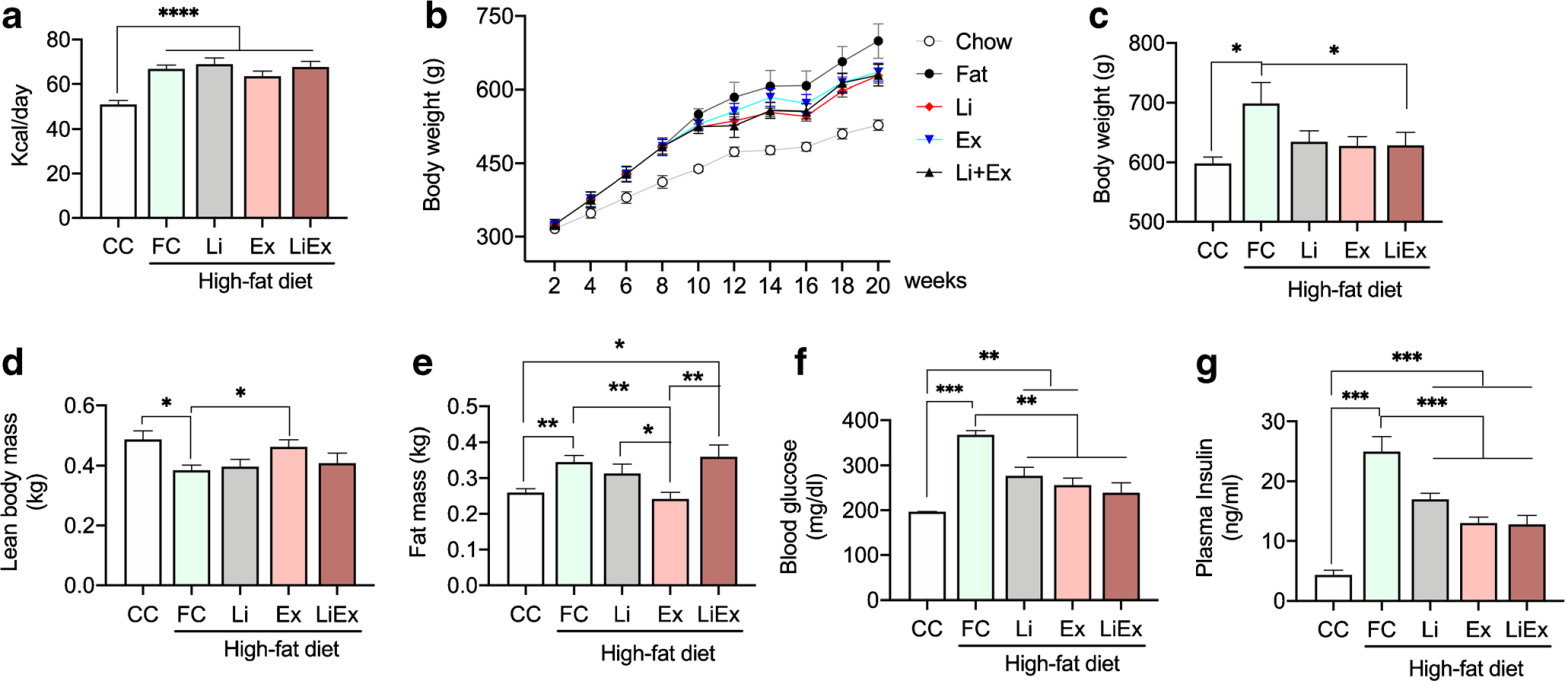
Long-term Li and exercise training ameliorates the metabolic phenotype of diet-induced obese (DIO) rats. Rats were fed a HFD for 8 weeks followed by treatment of Li and/or Ex for 12 weeks. **a** HFD increases calorie consumption. **b**, **c** Change in body weight. **d**, **e** Body composition. **f**, **g** Fasting blood glucose and insulin levels. Value are means ± SE, *p < 0.05, **p < 0.01, ***p < 0.001. CC; chow control, FC; fat control, Li; lithium, Ex; exercise, LiEx; lithium plus exercise

To investigate the effects of Li and low-volume exercise training on HFD-induced IR, rats were fed a HFD (42% fat-derived calories) or normal chow (chow) for 8 weeks. All HFD groups consumed higher kcal per day (Fig. 1a), and we found that the body weight (BW) in the FC group was the highest (*p* < 0.05) among all HFD groups (Li, Ex, and LiEx) (Fig. 1b, c). BW in the LiEx group was significantly (*p* < 0.05) lower than that in the FC group. Li and exercise decreased BW but not significantly (Fig. 1c). The DEXA scan revealed that the lean body mass (LBM) in HFD-fed mice was significantly (*p* < 0.05) lower than those fed chow, but exercise increased LBM more than that in the FC group (Fig. 1d), and HFD-fed mice showed significantly (*p* < 0.01) increased fat mass compared to those fed chow (Fig. 1e). Expectedly, the Ex group showed a decrease (*p* < 0.01) in fat mass compared to that in the FC group, but we found no change of fat mass in the LiEx group with FC (Fig. 1e). Interestingly, Li blocked exercise-induced loss of fat mass of DIO mice, we and previous studies did not find the reason [4, 6, 16]. A previous in vitro study showed that Li increases glucose transport rate in skeletal muscles; however, the level is not as sufficient as insulin or contraction effect [24]. Therefore, we speculated that combined treatment of Li with low-volume exercise for 12 weeks could adaptively increase glucose disposal, as shown by the insulin or contraction effect. We found that the blood glucose levels in the HFD-fed mice were significantly higher (*p* < 0.001) than those in the CC group, but blood glucose and insulin levels in Li, Ex, and LiEx groups were significantly (*p* < 0.01) lower than those in the FC group, despite the consumption of HFD (Fig. 1f, g). We thought the reason that fasting blood glucose and insulin levels in LiEx did not show significant effects compared to those in the Li group was because the levels were determined after 48 h of exercise training, and they continued to consume the HFD.

### Long-term Li and Ex training did not induce an adaptive increase in the levels of GLUT4 translocation factors in DIO rats

**Fig. 2:**
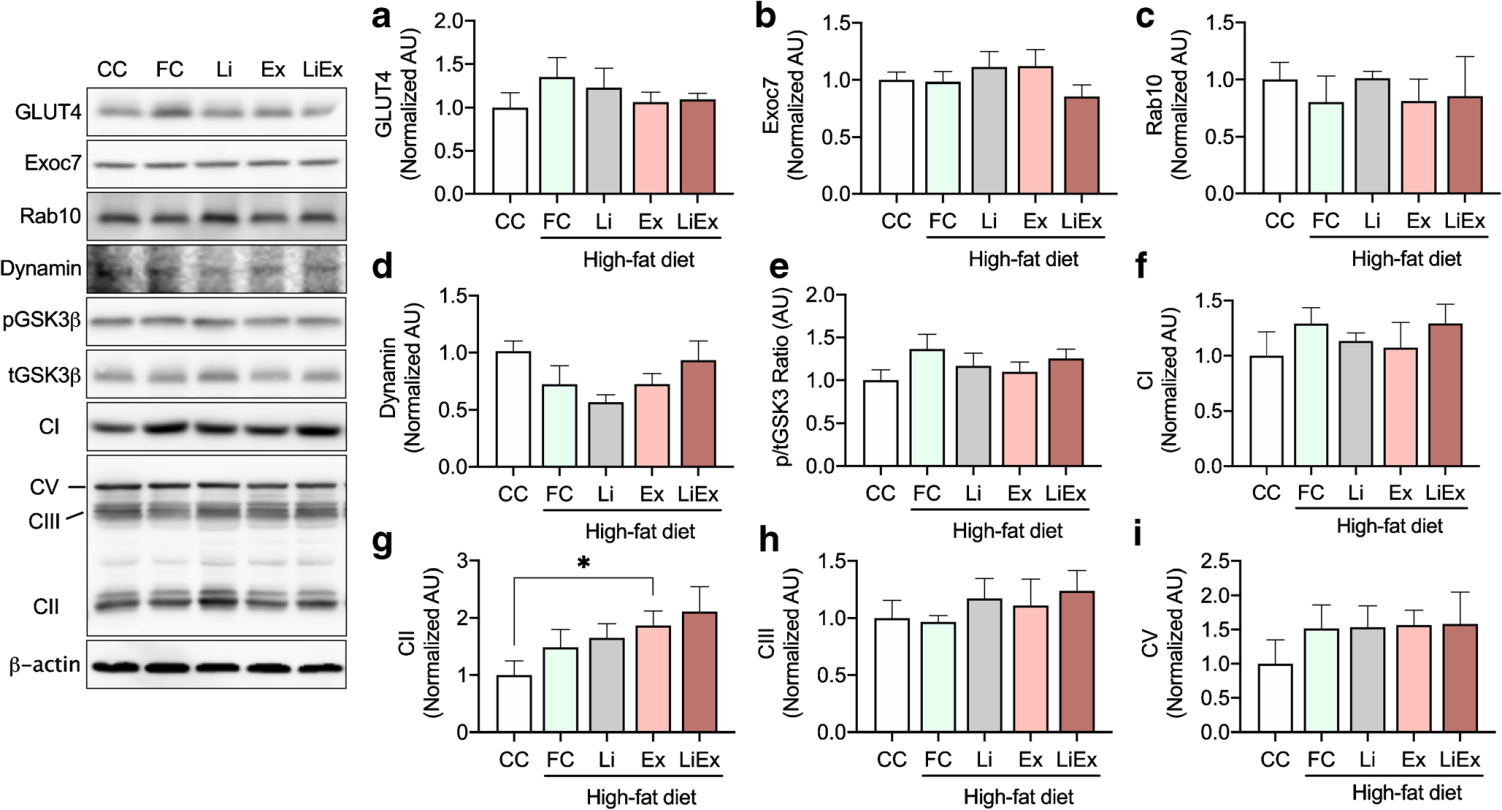
Long-term Li and exercise training did not significantly alter protein content in skeletal muscle. Protein content was determined by western blotting in muscle of rats. **a** GLUT4, **b** Exoc7, **c** Rab10, **d** Dynamin, **e** phospho-GSK3β ratio, and **f** mitochondrial complex (C) I, **g** CII, **h** CIII, **i** and CV were determined by western blotting (*n* = 10 muscles per group). Values are means ± SE, *p < 0.05. CC; chow control, FC; fat control, Li; lithium, Ex; exercise, LiEx; lithium plus exercise

We speculated that long-term treatment of Li with low-volume exercise for 12 weeks would increase the levels of various GLUT4 translocation factors; however, we found that Li and/or low-volume exercise for 12 weeks did not change GLUT4 content (Fig. 2a). Exocyst complex component 7 (Exoc7) [44] and RAS oncogene family (Rab10) [10] facilitate exocytosis of vesicles to the plasma membrane, the contents of these proteins were not altered by Li, exercise, and Li plus low-volume exercise for 12 weeks (Fig. 2b, c). Dynamin is a GTPase for the endocytosis of GLUT4 [2] and is not changed by Li, low-volume exercise, and Li plus low-volume exercise for 12 weeks (Fig. 2d). Glycogen synthase kinase 3β (GSK3β) is a serine/threonine protein kinase that mediates the glycogen synthesis, and we did not observe any effect of Li and low-volume exercise (Fig. 2e). Glucose and lipid metabolism are largely dependent on mitochondria, and we found that complex II mitochondria (CII) in the low-volume exercise group were significantly higher (*p* < 0.05) than that in the CC group (Fig. 2g); however, CI, CII, and CV were not changed by any of the treatments (Fig. 2f, h, i). These results indicate that either Li plus low-volume exercise did not induce adaptive response for translocation and activation of GLUT4, or it is possible that the dose of Li and exercise intensity were very low to induce an adaptive response of the skeletal muscle.

### Li facilitates glucose uptake induced by muscle contraction via glycogen breakdown

**Fig. 3:**
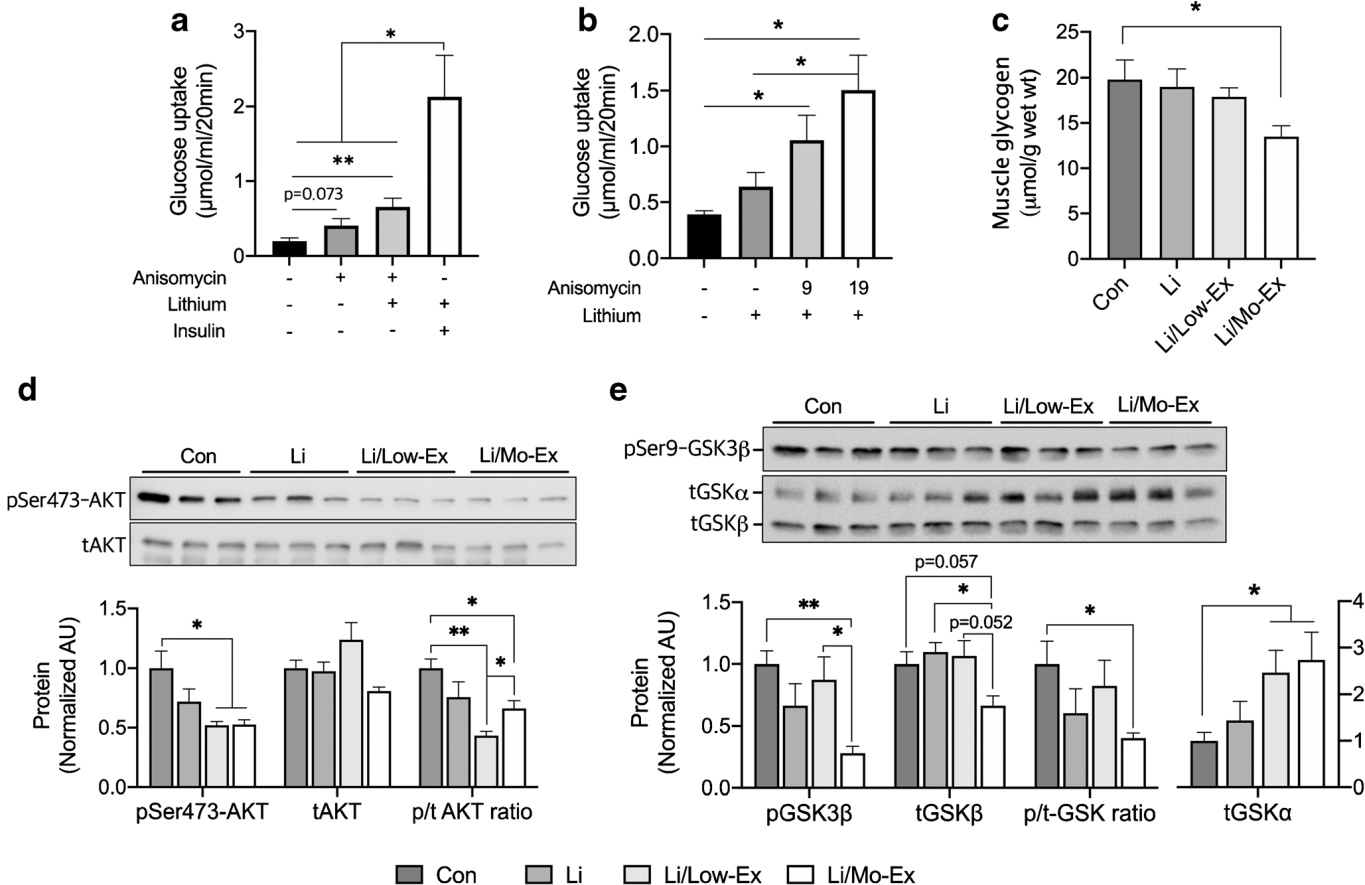
Li facilitates Ex or muscle contraction-induced glucose uptake. **a**, **b** Anisomycin, Li, and insulin was treated in isolated rats muscle, and determined glucose uptake (*n* = 6 muscles per group). **c**–**e** Mice were treated with Li along with Low-Ex or Mo-Ex. **c** Muscle glycogen level in mice was determined immediately after exercise or Li treatment. **d**, **e** AKT and GSK3β phosphorylation were determined by western blotting. **d** Effect of Li and acute exercise on AKT phosphorylation, **e** Effect of Li and acute exercise on GSK3β phosphorylation (*n* = 5 muscles per group). Value are means ± SE, *p < 0.05, **p < 0.01. Li; lithium, Low-Ex; low-volume exercise, Mo-Ex; moderate-volume exercise

Since muscle contraction induces glucose uptake in an insulin-independent manner and increases catabolism to support ATP, it consequently increases glycogen breakdown. A previous study showed that Li facilitates muscle contraction-induced glucose uptake [24], and Li and insulin reportedly increases glycogen synthesis [11]; therefore, it is interesting to understand the mechanism that of whether Li can facilitate glycogen synthesis or breakdown in a contracting muscle. We speculated that Li was involved in muscle contraction-induced glycogen breakdown and potentially facilitated glucose uptake in an insulin-independent manner, although Li with Ext did not induce proteins related to glucose uptake in skeletal muscle 48 h post-exercise (Fig. 2). First, we tested whether Li could enhance muscle contraction-induced glucose uptake in vitro, using anisomycin, which mimics muscle contraction [14] and slightly increases glucose uptake; however, Li treatment with anisomycin significantly increased (*p* < 0.01) glucose uptake, although this effect was significantly lower (*p* < 0.05) than that of Li treatment with insulin (Fig. 3a). We aimed to determine whether the effect of Li was affected by muscle contractile intensity and found that the effect of Li on muscle contraction-induced glucose uptake resulted in an increase depending on the muscle contraction intensity (Fig. 3b). Reportedly, AKT and GSK3β are linked to Li effects on glucose uptake [29]; therefore, we determined the effects of Li with low-intensity (12 m/min, Li/Low-Ex) for 20 min and moderate-intensity (20 m/min, Li/Mo-Ex) exercise for 40 min on AKT and GSK3β protein content. We found that Li with Mo-Ex significantly decreased muscle glycogen levels (Fig. 3c) and Li with Low- and Mo-Ex decreased pSer473-AKT levels, indicating that muscle contraction facilitates catabolism (Fig. 3d). Interestingly, only Li with Mo-Ex significantly decreased the phosphorylation and total amount of serine GSK3β, and these data also indicate that Li facilitates muscle contraction induced by catabolism (Fig. 3e). GSK3β phosphorylation in muscles that were subjected to Li and Mo-Ex treatments was significantly lower than that in the Con (*p* < 0.01) and Li/Low-Ex groups (*p* < 0.05). Interestingly, in the same group, total-GSK3β (tGSK3β) was significantly lower (*p* < 0.05) than that in the Li, Con (*p* = 0.057), or Li/Low-Ex (*p* = 0.052) groups, whereas tGSK3α in Li/Low-Ex and Li/Mo-Ex was significantly higher than Con group. Moreover, the GSK3β phosphorylation ratio was computed, and the phosphorylation ratio was found to be significantly lowered in the Li with Mo-Ex group compared to that in the Con (Fig. 3e). These data indicate that Li facilitates glucose uptake and glycogen breakdown induced by muscle contraction through block phosphorylation of Ser473-AKT and GSK3β.

### Li facilitates low dosage insulin-induced glucose uptake in T1DM mouse via Exoc7 and Ser473-AKT

**Fig. 4:**
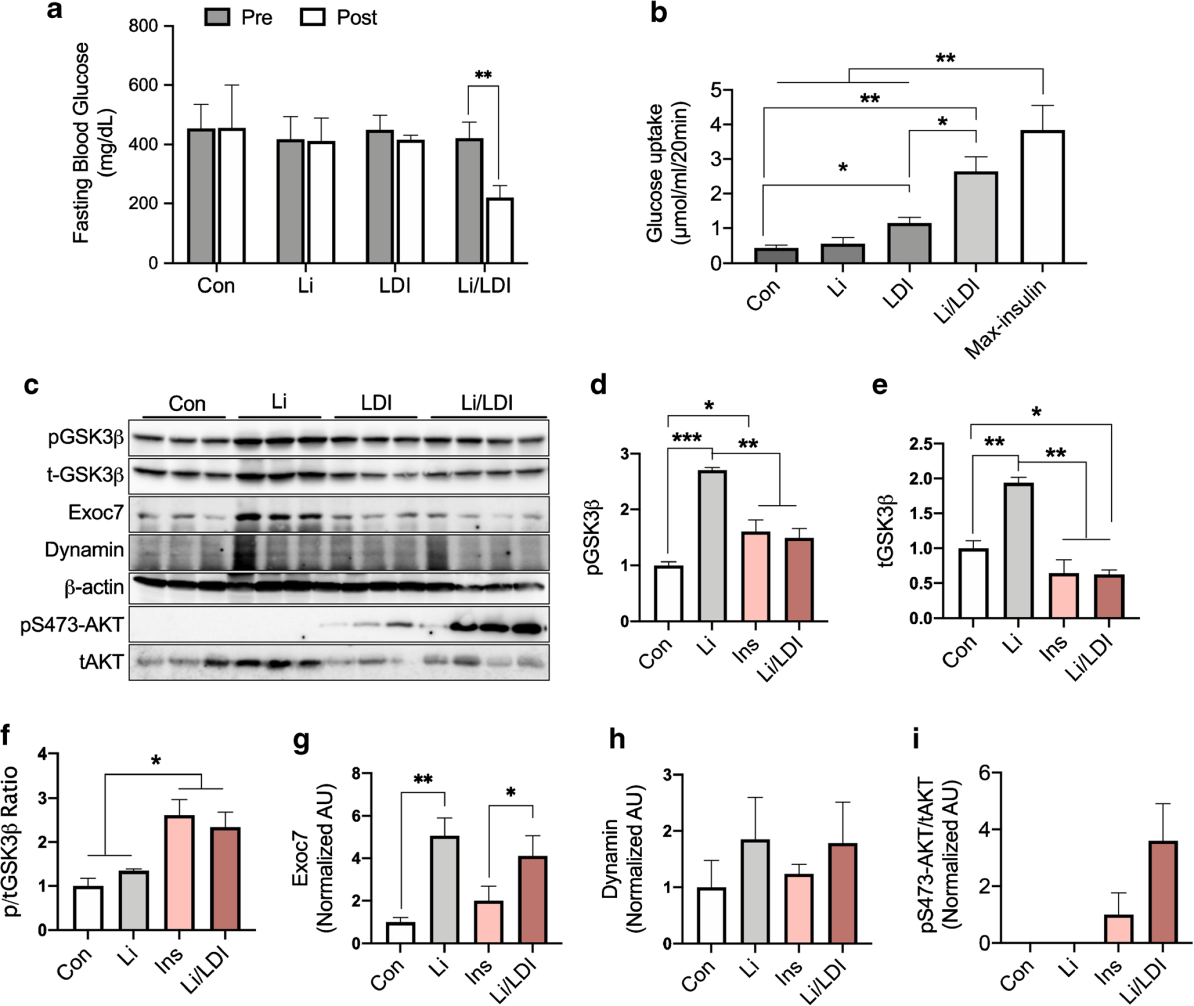
Li accelerates insulin-induced glucose uptake by enhancing AKT activation. **a**–**i** Mice were induced T1DM using STZ. **a** Fasting blood glucose level according to the treatment (*n* = 5 mice per group). **b** Muscle glucose uptake following treatment with Li or low dosage insulin (*n* = 5 muscle per group). **c**–**i** GSK3β phosphorylation (**d**), total GSK3β content (**e**), ratio of phosphorylation/total GSK3β (**f**), Exoc7 level (**g**), dynamin content (**h**), and ratio of S473 phosphorylation/total AKS (**i**) following Li and low dosage insulin (*n* = 5 muscles per group). Value are means ± SE, *p < 0.05, **p < 0.01, and ***p < 0.001. Con; control, Li; lithium, Ins; insulin, LDI; low-dose insulin

Since Li only exerted acute effects on glucose uptake (Fig. 3), low-volume exercise did not indicate the adaptive increase of Li-induced glucose disposal and uptake rate (Fig. 2), and thus, we speculated that Li could facilitate low dosage insulin-induced glucose disposal rate in T1DM, induced by streptozotocin (STZ). We found that the low dosage insulin or Li concentration used for patients with bipolar disorder did not affect fasting blood glucose levels in T1DM mice (Fig. 4a). However, Li plus low dosage insulin significantly increased (*p* < 0.01) glucose disposal in the blood of T1DM mice (Fig. 4a). The level of glucose uptake in the skeletal muscle induced by insulin was dependent on insulin concentration (Fig. 4b) and maximum insulin dosage resulted in an increase in an approximately 8-fold glucose uptake (Fig. 4b). However, low dosage insulin only increased approximately 2-fold in T1DM mouse skeletal muscle (Fig. 4b). We found that Li plus low dosage insulin additively increased glucose uptake in T1DM skeletal muscle, although Li alone could not induce glucose uptake (Fig. 4b). Treatment with low dosage insulin alone and Li plus low dosage insulin resulted in a higher increase in GSK3β phosphorylation ratio compared to treatment with Con or Li alone (Fig. 4c–i). We could not find any significance of dynamin induced by Li and/or insulin (Fig. 4h). Interestingly, acute Li consumption resulted in an approximately 5-fold increase in Exoc7 levels compared to those of the control in TA; however, low dosage insulin did not induce an increase in Exoc7 levels (Fig. 4g). Moreover, Li alone did not induce an increase in AKT phosphorylation and Li with insulin increased AKT activation by approximately 3.6-fold more than that by insulin (Fig. 4i). These effects contribute toward lower fasting blood glucose levels (Fig. 4a). Low-dosage insulin induced AKT phosphorylation (Fig. 4i), but it was not enough to reduce fasting glucose levels (Fig. 4a). Therefore, Li can facilitate glucose homeostasis via Exoc7-induced translocation of GLUT4 and insulin-mediated AKT activation in T1DM mice.

## Discussion

Although the effects of Li on the brain have been widely studied, most studies have focused on bipolar disorder and not on glucose uptake in skeletal muscle. A previous study indicated that Li increases glucose uptake in the skeletal muscle, although the capacity to take up glucose into muscles is lower than that of contraction and insulin stimulation [24]. Muscle is an important tissue that takes up ~ 80% glucose postprandial state; however, the mechanism underlying Li effect on glucose uptake in skeletal muscle has not been extensively studied and is largely unknown. Here, we studied whether exercise can activate GLUT4 induced by Li to take up glucose into skeletal muscle in obese mice, and if low-dose insulin can facilitate Li-induced glucose disposal in T1DM mice.

IR caused by HFD in the skeletal muscle decreases the capacity of blood glucose disposal in the postprandial state and increases the incidence rate of T2DM. Muscle contraction increases glucose uptake in an insulin-independent manner; however, there are issues pertaining to obtaining the benefits of exercise in the elderly or people who cannot exercise regularly. Generally, regular exercise training with 70% VO_2_ max improves insulin sensitivity, GLUT4 levels, and mitochondrial enzyme content in the skeletal muscle. We speculated that Li could induce the adaptive response of skeletal muscle, even with low-intensity exercise and duration, and this adaptive response can assist patients who require better glucose homeostasis. However, no effects of GLUT4 translocation, glucose uptake, and adaptive response induced by Li with Low-Ex training were found 48 h post-exercise. Although Li, Ex, and LiEx groups were fed an HFD, loss of body weight in the rats of these groups might reduce fasting glucose levels. We did not demonstrate insulin responsiveness in the skeletal muscle. Since it is possible that insulin sensitivity in muscle was improved by long-term Li, Ex, and LiEx treatment, we cannot exclude the possibility that improved insulin sensitivity induces lower fasting glucose levels. Thus, further study is warranted.

Even though Li did not induce an adaptive response of skeletal muscle following low-volume exercise, Li enhanced muscle contraction-induced glucose uptake in a contraction intensity-dependent manner. Interestingly, Ser473-AKT and Ser9-GSK3β were dephosphorylated in an exercise volume-dependent manner. This observation provides new insights into how Li facilitates muscle contraction-induced glucose uptake, because the dephosphorylation of Ser473-AKT and Ser9-GSK3 blocks the metabolic flow of glycogen synthesis, which can facilitate glycogen breakdown and support pyruvate for muscle contraction. Moreover, a previous study has shown that GSK3β is sensitive to the chelation of free Mg^2+^ by ATP and is gradually decreased when ATP concentrations exceed that of Mg^2+^ [33]. Thus, the reduction of ATP concentration by muscle contraction increases GSK3β activity (dephosphorylation), which decreases glycogen synthesis by glycogen synthase (GS) activation and facilitates glycogen breakdown to replenish ATP. Li did not directly affect glycogen levels in the skeletal muscle, and the reduction of Ser473-AKT and Ser9-GSK3β phosphorylation by Li may facilitate glycogen breakdown. The rate of carbohydrate oxidation depends on exercise intensity [32, 42], and muscle contraction increases glycogen breakdown [34] to replenish pyruvate. As shown in a previous study, muscle contraction stimulates glucose uptake, and the ratio depends on glycogen breakdown based on contraction intensity [3, 25].

Interestingly, Li ingestion along with acute exercise increased tGSK3α, whereas it decreased tGSK3β, the phosphorylation rate of GSK3β will inevitably be low in muscles with low tGSK3β content, and this may facilitate the inactivation of GS, which cannot carry out glycogen synthesis. Previous studies have shown that GKS3β inhibition improves glucose uptake [7]. Moreover, glycogen levels after muscle contraction show a positive correlation with glucose uptake level and glycogen breakdown induced by muscle contraction in skeletal muscles may improve insulin sensitivity at the resting state [23]. Therefore, Li enhances glucose uptake induced by muscle contraction by increasing GLUT4 translocation and glycogen breakdown.

T1DM patients require daily injections of insulin for life, and trials to amplify the delivery and capacity of insulin are continuously required for ensuring the health of T1DM patients [35]. Therefore, we speculated that Li could be used to support the insulin effect and demonstrated that Li with insulin resulted in an increase in glucose uptake by approximately 6-fold more than that of the control and in an increase 2.3-fold more than the low-dose insulin that mimicked lower absorption of insulin, although the effect was lower than that of the maximum insulin dosage. Moreover, low-dose insulin (or lower absorption) could not decrease fasting blood glucose levels; however**,** treatment of Li along with insulin improved the homeostasis of blood glucose in T1DM mice. Recently, Exoc7 gene deletion in adipocyte has been shown to inhibit insulin-stimulated GLUT4 exocytosis [44], and we observed in the present study that Li increased GLUT4 exocytosis via Exoc7 and glucose uptake. However, since Li alone did not increase AKT activation, it appears that just increasing Exoc7 by Li alone is not sufficient to take much glucose up for T1DM mice as insulin treatment. A previous study has shown that Li inhibits the internalization of GLUT4; however, unlike insulin and muscle contraction, Li cannot highly activate GLUT4 to take up glucose into the muscle [24]. Thus, Li cannot be developed as an alternative to insulin. However, we found that Li can be a transitional alternative candidate for T1DM patients when using low-dose insulin. Li along with low-dose insulin increases glucose uptake and Ser473-AKT activation more than insulin alone and decreases glucose levels in the blood. AKT activation induces GLUT4 translocation and stimulates glucose uptake [36]. Therefore, Li enhances low-dose insulin-induced glucose uptake via GLUT4 translocation and S473-AKT activation in T1DM mouse skeletal muscle. However, Li alone did not activate Ser473-AKT; thus, the mechanisms through which Li synergistically increases AKT activation along with insulin warrant additional studies.

Taken together, the effect of Li on increasing GLUT4 translocation into the plasma membrane can enhance glucose uptake by muscle contraction-induced glycogen breakdown. Furthermore, AKT activation by insulin can activate GLUT4 translocated by Li and facilitate glucose uptake more than that by low-dose insulin in T1DM skeletal muscle. Li regulates GSK3β activation level according to glycogen availability in contracting or resting muscles. Thus, the elderly, people with problems in exercising regularly, or T1DM patients require a transitional alternative to remain healthy, and Li could serve as a candidate therapeutic target and be relatively easily developed, as Li is already used to treat other disorders.

## Supplementary Information

ESM 1 (DOCX 700 kb)

## Data Availability

The data that support the findings of this study are available from the corresponding author upon reasonable request.

## Declarations

The study design was approved by the Animal Experiment Ethics Committee of Daegu Technopark BioHealth Convergence Center (BHCC-IACUC-2018-01).
