# Supplementary material for: Lithium enhances exercise-induced glycogen breakdown and insulin-induced AKT activation to facilitate glucose uptake in rodent skeletal muscle
Source: Pflugers Arch. 2021 Mar 3;473(4):673–82. doi: 10.1007/s00424-021-02543-0 (PMC8049887; doi:10.1007/s00424-021-02543-0)
Supplement: Supplementary file 1 — (DOCX 700 kb) [file 424_2021_2543_MOESM1_ESM.docx]

**Supplement figure 1.**


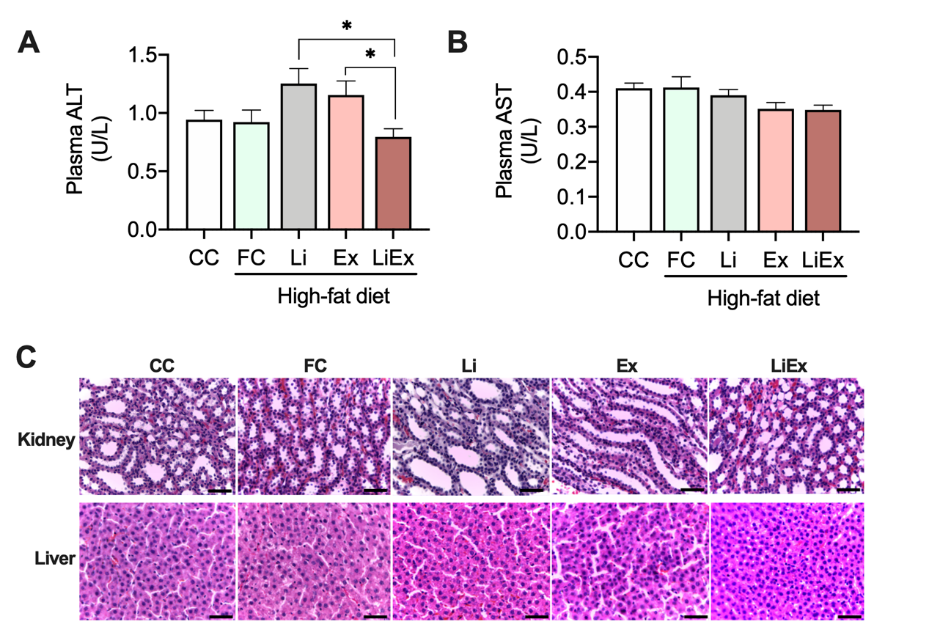


**Figure S1**. Long-term Li and exercise training does not induce toxicity in the kidney and liver. Rats were fed a HFD for 8 weeks followed by treatment of Li and/or Ex for 12 weeks. Toxicity test of Li was performed using (**A**,**B**) fasting plasma ALT and AST levels and. H&E staining in the kidney and liver **(C)**. Value are means ± SE, *p<0.05, Scale bar, 50 µm. CC; chow control, FC; fat control, Li; lithium, Ex; exercise, LiEx; lithium plus exercise.

**Long-term Li treat did not induce toxicity in the kidney and liver of DIO rats**

Li is commonly used as a medication to treat several mental health conditions. Although we used typical doses for patients, since oral administration of Li affects body composition, we tested the toxicity of Li doses for rats and found that the plasma alanine aminotransferase (ALT) levels in the Li and Ex groups were significantly (p<0.05) higher than those in the LiEx group, but the levels were within the normal range (Fig. S1A). There was no difference in the aspartate aminotransferase (AST) levels (Fig. S2B) and histological matters among all the groups (Fig. S2C).
